# Experimental dataset for the macro-scale compression of Norway Spruce perpendicular to grain direction

**DOI:** 10.1016/j.dib.2021.107742

**Published:** 2021-12-22

**Authors:** Tito Adibaskoro, Wojciech Sołowski, Simo Hostikka

**Affiliations:** Aalto University School of Engineering, Department of Civil Engineering, Rakentajanaukio 4A, 02150 Espoo, P.O. Box 12100, Aalto 00076, Finland

**Keywords:** Load-displacement history, Orthotropic, Post-failure, Quasi-static, Transverse, Universal testing machine, Video, Wood material

## Abstract

This article presents load and displacement data of *Norway Spruce* quasi-static compression test as well as video recordings of the experiment using the *Universal Testing Machine* (UTM). The specimens are 4 cm × 4 cm × 8 cm clear-wood cuboids with grain direction perpendicular to and loading direction parallel to the long axis. Due to the radially-arranged annual ring at such scale of the specimens, the plane of interest features significantly-varying orientation of the weak axes, namely the *radial* (R) and *tangential* (T) directions. A hemispherical knee joint underneath the specimen were fine-adjusted under preload before each experiment to ensure evenly-distributed load. Additionally, grids of 5 mm spacing were drawn on the plane of interest for better clarity of the deformed shape. Both load and displacement history recorded by the UTM as well as the deformation process recorded as video may provide valuable information for validation of wood material models or simulation methods with wood material implementations.

## Specifications Table

**Table:** 

Subject	Civil and Structural Engineering
Specific subject area	Experimental study of wood material behaviour in transverse direction
Type of data	Table (Excel) Video
How the data were acquired	The experiment was conducted using the Zwick RK 250/50 *Universal Testing Machine* (UTM) with HBM U2A load cell set at 200 kN capacity. The UTM was equipped with a hemispherical knee joint placed underneath the specimen. The UTM records load and displacement history at 50 Hz. Videos were recorded with Canon EOS 5DS R mounted on a tripod. Experimental procedure follows SFS EN 408:2010+A1:2012 [1]. Specimen weights and dimensions are measured with a digital scale and a digital calliper respectively.
Data format	Raw
Description of data collection	Samples were clear *Norway Spruce* timbers cut into 4 cm × 4 cm × 8 cm pieces in a way such that each has the growth ring centre located around one edge. After they were cut, the specimens were kept in a climate room at 20 °C and 65% relative humidity before the experiment.
Data source location	• Institution: Aalto University School of Engineering • City/Town/Region: Espoo • Country: Finland • Latitude and longitude: 60.18744904922521, 24.83140920338534
Data accessibility	Repository Name: Zenodo Data Identification Number: 5721130 Direct URL to data: https://doi.org/10.5281/zenodo.5721130
Related research article	T. Adibaskoro, W. Sołowski and S. Hostikka, “Multi-Surfaced Elasto-Plastic Wood Material Model in Material Point Method,” *International Journal of Solids and Structures,* 2021, doi:10.1016/j.ijsolstr.2021.111333.

## Value of the Data

• The data provides insight on the failure mode of symmetrical clear wood specimen with asymmetrical arrangement of material orientation.
• Numerical studies with wood material that differentiates transverse direction into radial (R) and tangential (T) (via e.g. macroscopic material or multiscale modelling) may use the data for validation.
• The data can be reused in inverse-problem studies to predict radial (R) and tangential (T) wood material behaviour.

## 1 Data Description

### 1.1 “Load-Disp history.xlsx”

The excel file contains load and displacement history recorded by the Universal Testing Machine (UTM) at 50 Hz. The specimens are renamed to match the nomenclature of the supported research article.

### 1.2 “Specimen1.mp4”

Recording of the compression test of specimen 1 (nomenclature matched with “Load-Disp History.xlsx”). Recording started before loading process commenced and ended after reaching the 20 mm displacement target.

### 1.3 “Specimen2.mp4”

Recording of the compression test of specimen 2 (nomenclature matched with “Load-Disp History.xlsx”). Recording started before loading process commenced and ended after reaching the 20 mm displacement target.

### 1.4 “Specimen3.mp4”

Recording of the compression test of specimen 3 (nomenclature matched with “Load-Disp History.xlsx”). Recording started before loading process commenced but unfortunately ended prematurely near the beginning.

### 1.5 “Specimen Weights and Dimensions.xlsx”

The excel file contains weights and dimension measurements of all the three specimens, with specimen nomenclature following that in "Load-Disp History.xlsx".

## 2 Experimental Design, Materials and Methods

### 2.1 Specimen

**Fig. 1:**
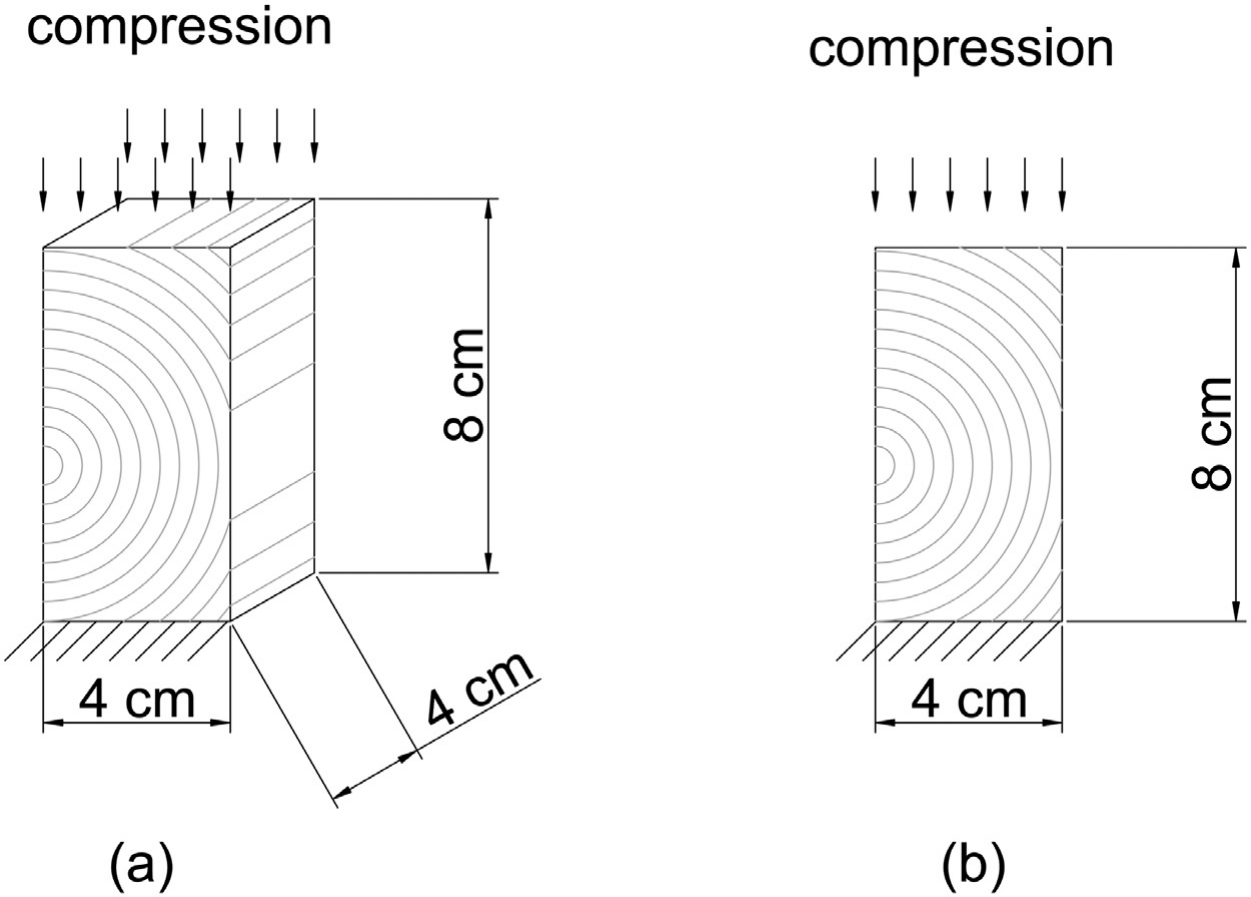
The compression test specimen is 4 cm × 4 cm × 8 cm cuboid-shaped clear Norway Spruce wood, cut such that the centre of the annual rings is located near the edge (a); the experiment's plane of interest is the one which cross-section features the radially-arranged annual ring [3].

**Fig. 2:**
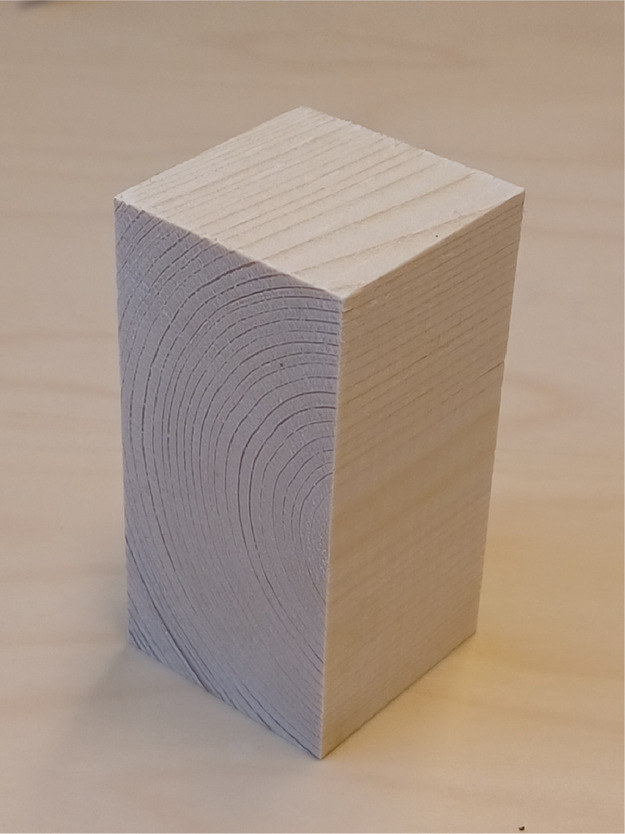
An example of defect-free sample cut according to the size and material orientation target illustrated in Fig. 1(a).

The tested specimens are three 4 cm × 4 cm × 8 cm *Norway Spruce* clear wood cut from beams in a way so that the centre of the annual ring is situated near one edge of the specimen. Fig. 1(a) illustrates how we aim to cut the specimens in terms of dimension and position of the centre of the annual ring, while Fig. 1(b) shows the plane-of-interest in this experiment. An example of a cut sample according to the said specifications is shown in Fig. 2. For the particular tested specimens, Fig.s 3 (a)-(c) show their state before the experiment. Of all the produced samples, about half were discarded due to the presence of wood imperfections. Defect-free samples are stored in climate room of 20°C temperature and 65% relative humidity for about 1 month to approach 12% moisture content (see [2]).

**Fig. 3:**
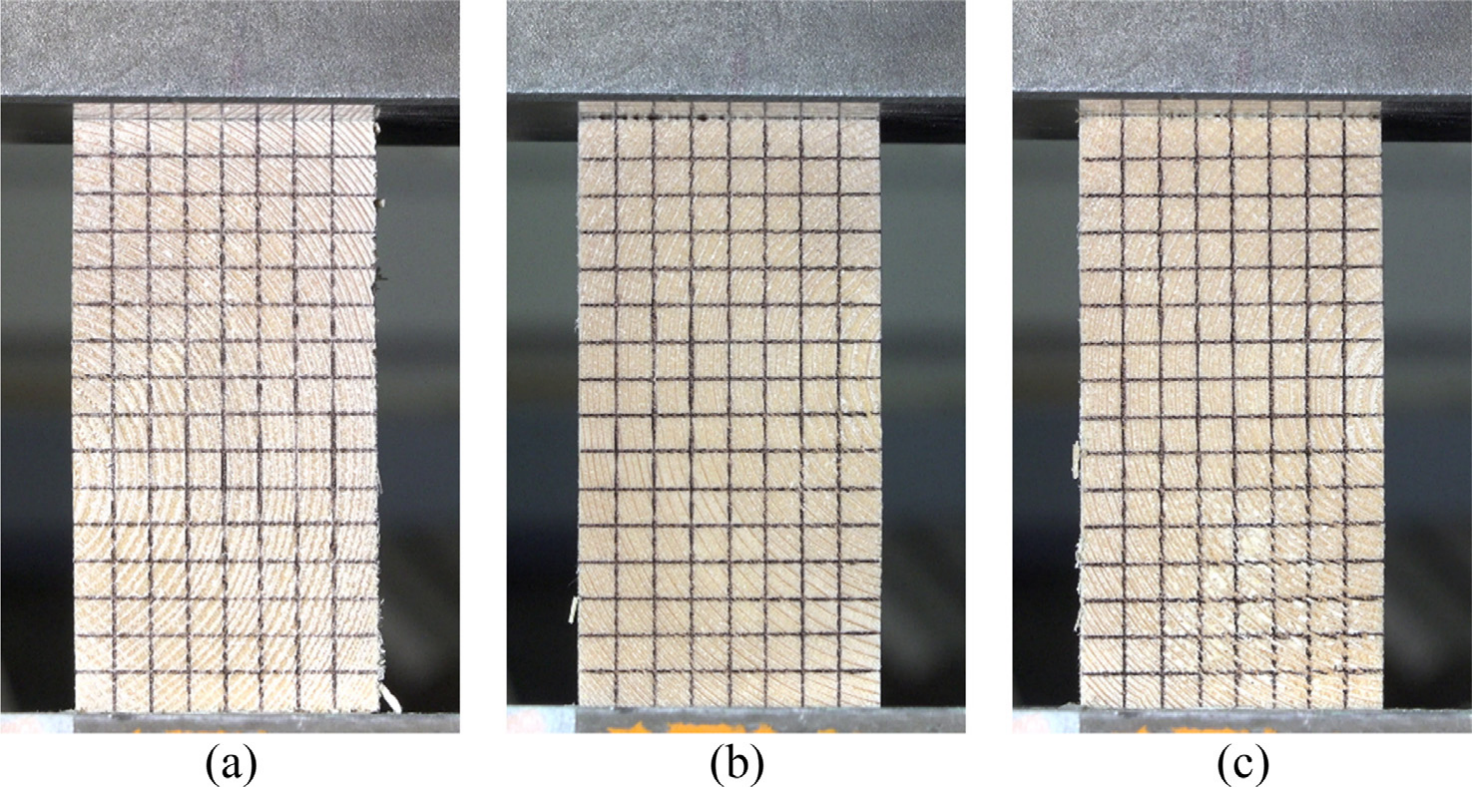
Specimens 1 (a), 2 (b), and 3 (c) just before experiment starts [3].

**Fig. 4:**
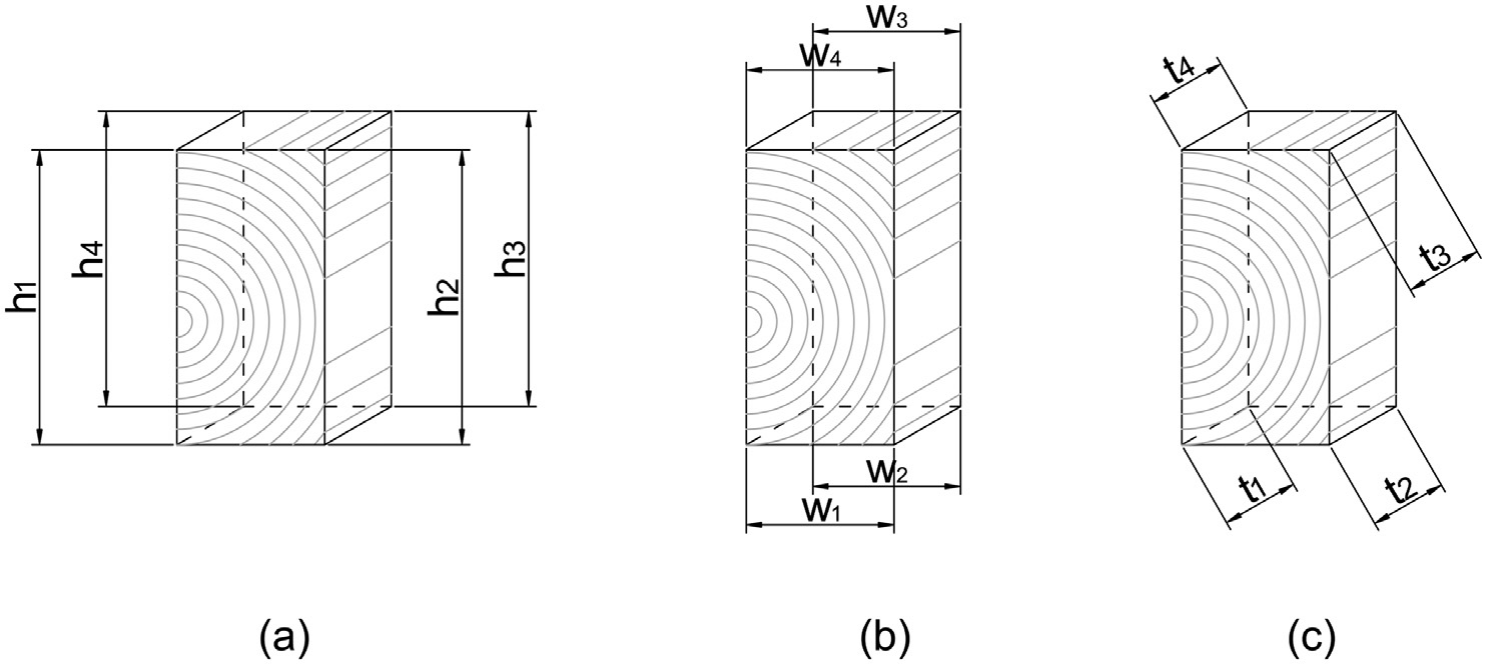
The reported dimensions of height (a), width (b), and thickness (c) are averaged from measurements at the four edges with a caliper.

All specimens are weighed before the experiment. Additionally, we measure the specimen's dimensions to ensure good approximation to the aforementioned target sizes. Table 1 shows the weights and dimensions of specimens 1-3. The dimensions are reported by averaging calliper measurements from all 4 edges (see Fig.s 4 (a), (b), and (c) for height (h), width (w), and thickness (t) respectively). Table 2 shows the measurements of those four edges for each dimension. The data presented in both Tables 1 and 2 and Fig. 4 are included in *Specimen Weights and Dimensions.xlsx*.

**Table 1:** Weight and measured dimensions of the specimens before experiment.

Specimen	Weight (g)	Dimension		
		h (mm)	w (mm)	t (mm)
1	65.48	80.85	40.89	39.85
2	69.22	80.76	40.83	40.80
3	66.26	80.76	40.92	40.33

### 2.2 Procedure

**Fig. 5:**
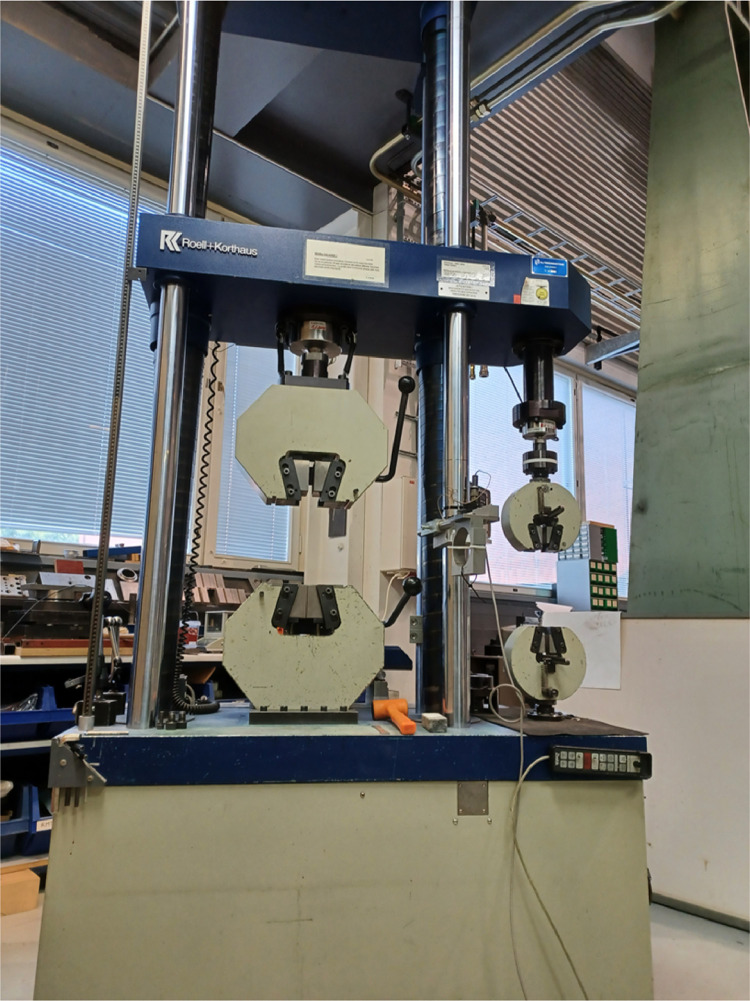
The Zwick RK 250/50 Universal Testing Machine (UTM) applies compressive force while recording the load and displacement history at 50 Hz

The compression experiment utilizes the Zwick RK 250/50 *Universal Testing Machine* (UTM) shown in Fig. 5, which provides displacement-controlled compression and records the data. The procedure follows the guidelines of SFS EN 408:2010+A1:2012 [1] for wood compressive strength test perpendicular to grain direction. Before every compression test, the knee joint was fine-adjusted with rubber hammer under preload to ensure even compression throughout the specimen. After locking the knee joint, compression test proceeded with displacement-controlled compression loading up to 20 mm of displacement, which allows plenty of compression for observation of the post-failure behaviour. The loading rate is determined by preliminary tests before the actual experiment.

**Table 2:** Measured specimen dimensions from all four edges each.

Specimen	h (mm)				w (mm)				t (mm)			
	h_1_	h_2_	h_3_	h_4_	w_1_	w_2_	w_3_	w_4_	t_1_	t_2_	t_3_	t_4_
1	80.94	80.86	80.86	80.75	40.92	40.86	40.88	40.90	39.80	39.98	39.81	39.80
2	80.76	80.64	80.71	80.93	40.92	40.80	40.83	40.76	40.80	40.83	40.72	40.83
3	80.90	80.90	80.82	80.43	40.91	40.92	40.92	40.92	40.26	40.37	40.32	40.37

The European Standard [1] requires compression test to reach maximum load within the range of (300 ± 120) s. Note that the definition of maximum load in said literature does not necessarily mean the highest load achieved throughout the test, but rather determined by the iterative 1%-offset approach (see [1]). Preliminary tests revealed 0.5 mm/minute to be the ideal loading rate for meeting the time requirement while convenient to work with. The chosen loading rate resulted in specimens 1, 2, and 3 reaching maximum load at 240.65 s, 238.05 s, and 238.36 s, well within the allowable range.

**Fig. 6:**
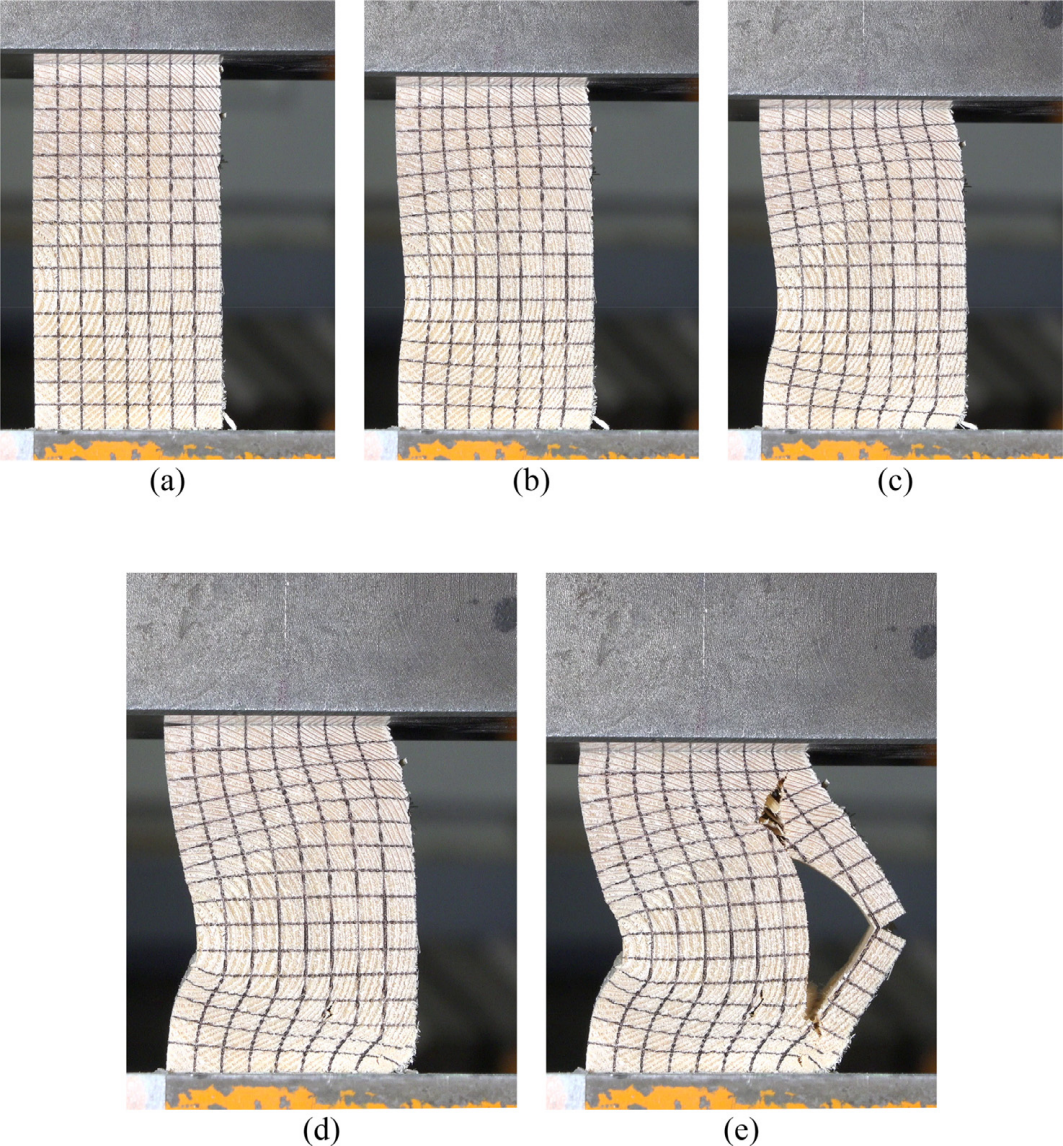
The undeformed shape of specimen 1 (a) and deformed shape at 5 mm (b), 10 mm (c), 15 mm (d), and 20 mm (e) of displacement [3].

**Fig. 7:**
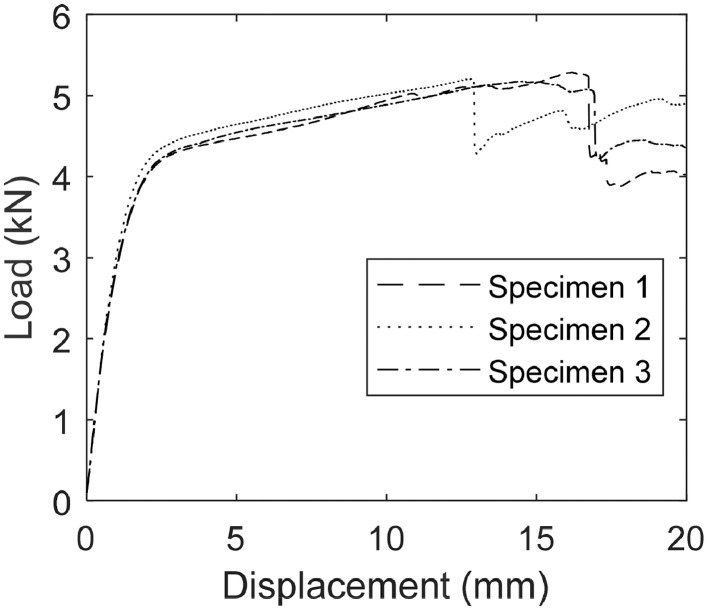
The load-displacement curves of specimens 1-3 from data presented in *Load-Disp History.xlsx*[3].

Additional floodlights provided even illumination of the video-recorded surface. We chose DC-powered lights to eliminate the possibility of flickering due to AC polarity reversal. Fig. 6 shows the deformed shape of specimen 1 throughout the experiment at various displacements taken from the video frames of *Specimen1.mp4*. The resulting load-displacement curves from load and displacement history data in *Load-Disp History.xlsx* is plotted in Fig. 7.

## Ethics Statement

This work involves neither human nor animal subjects.

## CRediT Author Statement

**Tito Adibaskoro:** Conceptualization, Methodology, Investigation, Writing-Original draft preparation. **Wojciech Sołowski**: Conceptualization, Supervision, Writing-Reviewing and Editing. **Simo Hostikka:** Supervision, Writing-Reviewing and Editing, Funding acquisition.

## Declaration of Competing Interest

The authors declare that they have no known competing financial interests or personal relationships that could have appeared to influence the work reported in this paper.

## Appendix Supplementary materials

Image, application 1
